# Prediction of motion artifacts caused by translation in handheld laser speckle contrast imaging

**DOI:** 10.1117/1.JBO.28.4.046005

**Published:** 2023-04-18

**Authors:** Ata Chizari, Wilson Tsong, Tom Knop, Wiendelt Steenbergen

**Affiliations:** University of Twente, Technical Medical Centre, Faculty of Science and Technology, Biomedical Photonic Imaging Group, Enschede, The Netherlands

**Keywords:** analytical models, biomedical optical imaging, computer simulation, Doppler effect, laser speckle contrast imaging, motion artifacts, model-driven development, numerical analysis

## Abstract

**Significance:**

In handheld laser speckle contrast imaging (LSCI), motion artifacts (MA) are inevitable. Suppression of MA leads to a valid and objective assessment of tissue perfusion in a wide range of medical applications including dermatology and burns. Our study shines light on the sources of these artifacts, which have not yet been explored. We propose a model based on optical Doppler effect to predict speckle contrast drop as an indication of MA.

**Aim:**

We aim to theoretically model MA when an LSCI system measuring on static scattering media is subject to translational displacements. We validate the model using both simulation and experiments. This is the crucial first step toward creating robustness against MA.

**Approach:**

Our model calculates optical Doppler shifts in order to predict intensity correlation function and contrast of the time-integrated intensity as functions of applied speed based on illumination and detection wavevectors. To validate the theoretical predictions, computer simulation of the dynamic speckles has been carried out. Then experiments are performed by both high-speed and low-framerate imaging. The employed samples for the experiments are a highly scattering matte surface and a Delrin plate of finite scattering level in which volume scattering occurs.

**Results:**

An agreement has been found between theoretical prediction, simulation, and experimental results of both intensity correlation functions and speckle contrast. Coefficients in the proposed model have been linked to the physical parameters according to the experimental setups.

**Conclusions:**

The proposed model provides a quantitative description of the influence of the types of illumination and media in the creation of MA. The accurate prediction of MA caused by translation based on Doppler shifts makes our model suitable to study the influence of rotation. Also the model can be extended for the case of dynamic media, such as live tissue.

## 1 Introduction

Four decades ago, a technique was developed for the measurement of spatiotemporal blood flow variations in the microcirculation by the analysis of laser speckle.^1^ Since then, this technique, known as laser speckle contrast imaging (LSCI),^2^^–^^5^ has advanced in different aspects, such as enhancement of spatial resolution,^6^ processing time,^7^ and quantitative prediction of blood flow.^8^^–^^11^ When coherent light encounters an optically rough surface or propagates through a scattering medium such as tissue, an interference pattern called speckle is observed on an image screen. Movements of the medium or scattering particles inside it cause formation of dynamic speckles. Imaging of dynamic speckles with a limited exposure time results in speckle pattern images of reduced contrast. The speckle contrast is a measure of motion on or inside the illuminated target: lower speckle contrast corresponds to higher movement.^3^

LSCI covers a wide range of clinical applications,^12^ including psoriasis,^13^^,^^14^ diabetic foot,^15^ and blood flow monitoring during surgery (intraoperative).^16^^,^^17^ Operated in handheld mode, LSCI brings convenience for both patients and clinical staff.^18^ However, involuntarily movements of the subject or the operator result in movement artifact, that is spatial shifting of the consecutive images (off-axis imaging) as well as extra blurring of individual speckle patterns. The former can be overcome by movement detection and image registration.^19^ The latter is more complicated and has been addressed so far in different ways: (i) motion stabilization using a gimbal mount as a prevention of motion artifacts (MA) due to operator motion^20^ and (ii) capturing speckle patterns from an opaque surface mounted in the field of view during the measurements. The perfusion measured on the opaque surface was used either to choose a number of movement-artifact-free frames^21^ or to be removed from the perfusion values measured on tissue as a compensation of MA.^22^^–^^25^ Altogether, a fundamental model to explain the underlying reason but also to predict MA is missing.

In a previous work, we studied MA, defined by the drop in speckle contrast due to the relative movements of the device and the object of measurement, due to the translation, rotation, and tilt of wavefronts. We found that the lower the scattering level of the medium is, the higher the drop of the speckle contrast is.^26^ In another study, we showed that use of planar or spherical waves for illumination instead of conventional scrambled waves made by glass (engineered) diffusers is a hardware-based and straightforward approach to decrease the MA.^27^

Then we carried out a comparison between handheld and mounted measurements on psoriasis patients. Results suggested that although the pair of mounted and handheld measurements were in agreement on a visual basis, the difference in lesion perfusion between mounted and handheld measurements was on-average statistically significant.^28^ This statistically significant difference caused by the movement-induced perfusion in handheld measurements may limit certain applications of handheld LSCI.

In this work, we use the concept of optical Doppler effect^29^ to predict speckle contrast drop due to translation and for solid objects. This is a first step in modeling MA due to linear motion and rotation when measuring on objects with internal motion, such as perfused tissue. This paper presents a theoretical model that describes part of the MA, namely translation on a highly scattering surface (matte paint) and a tissue-level scattering object (Delrin) validated by simulation of dynamic speckles and experiments.

## 2 Model of the Optical System and Speckle Contrast Generation

### 2.1 Optical Doppler Shifts Induced by Translation of a Solid Object

**Fig. 1:**
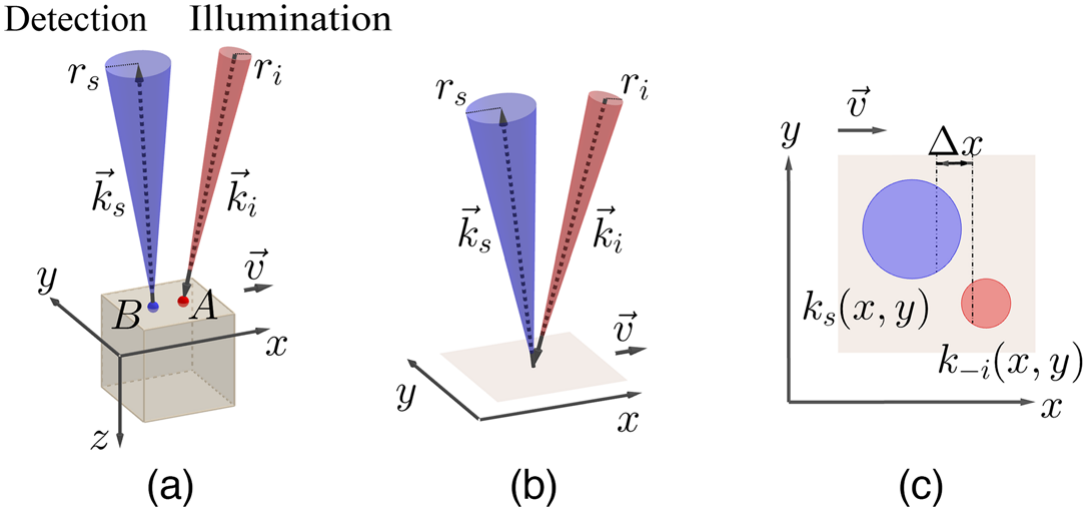
Schematic diagram of the system model reprinted with permission from Ref. 30. Linear translation of a solid object along the x–y plane in the x direction. Illumination and detection on a solid object of (a) finite and (b) very high scattering levels. (c) Mapping of the bases of the illumination and detection cones on the object’s surface (x–y plane).

Consider a solid object of finite scattering level moving with a constant velocity $\overset{\to }{v}$ along the x−y plane in the x direction [see Fig. 1(a)]. The object is illuminated over a large area by a coherent light source. For a given point A on the object’s surface, a cone of base radius $r_{i}$ is formed that is defined by the range of incoming wavevectors $\vec{k_{i}}$. Assuming a lens collecting scattered light at point B located at the object’s surface, a cone of base radius $r_{s}$ defined by the range of wavevectors $\overset{\to }{k_{s}}$ will be formed. The collecting lens will conjugate the point B to a point on the imaging screen of a detector causing time varying intensity I(t) containing a range of frequencies. The core of our model is that these frequencies are assumed to be dictated by the optical Doppler effect as^29^

$$\omega_{D}=\overset{\to }{v}.(\overset{\to }{k_{s}}-\overset{\to }{k_{i}}).$$ (1)

The type of illumination determines the spread of $\vec{k_{i}}$ and, therefore, the size of $r_{i}$. For scrambled waves made by a diffuser, the $r_{i}$ takes a nonzero value. For spherical waves or plane waves, there is only a single $\vec{k_{i}}$ on A, which leads $r_{i}$ to be zero. Notice that for spherical waves, the single $\vec{k_{i}}$ is position dependent and for scrambled waves, the orientation of the cone of $\vec{k_{i}}$ is position dependent. The openness of a diaphragm located at the base of a detection cone controls the spread of $\vec{k_{s}}$ and consequently the size of $r_{s}$. If the scattering medium is of finite scattering level, then for each detection point B on the object’s surface, the scattered light originally entering the medium at a range of positions around B contributes to the observed Doppler shift introduced in Eq. (1). This range depends on the type of illumination and the optical properties of the medium. Note that the relative speed between the pair of illumination-detection and scattering object at each moment forms $\vec{v}$ in Eq. (1); thus either of the two can be subjected to translational movements.

#### 2.1.1 Highly scattering medium

Consider the scattering medium shown in Fig. 1(a) to be of high scattering level such that no light penetrates into the medium (e.g., a white matte surface). Then A and B will coincide, and for each observation point B, only the incoming wavevectors to B and the outgoing wavevectors from B will contribute to creation of Doppler shifts on the conjugated point on the detector. Now the problem narrows down to a simplified geometry as shown in Fig. 1(b). If the bases of illumination and detection cones are mapped on the x−y plane and assuming that the angles between the normal to the surface and both $\vec{k_{i}}$ and $\vec{k_{s}}$ are relatively small, then the begin points of $\vec{k_{-i}}(x,y)$ and the end points of all $\vec{k_{s}}(x,y)$ occupy a circular region with radius $r_{i}\;$ and $r_{s}$, respectively, as shown in Fig. 1(c). Since the object moves in the x direction, the consequence of Eq. (1) is that all the wavevectors differences indicated by $\Delta x=x_{2}-x_{1}$ [see Fig. 1(c)] will create the same Doppler shift. The intensity fluctuations are caused by mixing light at various Doppler shifts, whereas light with a single Doppler shift does not yield an intensity fluctuation by itself. The frequencies of the fluctuations are caused by the variation of the Doppler shifts but not the absolute Doppler shifts. Consequently, the absolute tilt angles of the wavevectors are not of importance but the wavevector spreads that are modeled by $r_{i}$ and $r_{s}$.

From Eq. (1), a single Doppler shift as a function of the wavevector difference can be written as

$$\omega_{D}(\Delta x)=\frac{2\pi V}{\lambda }[l_{s}(x_{2}),l_{-i}(x_{1})],$$ (2)

where V is absolute speed, λ is the wavelength of the light source, and $l_{s}(.)$ and $l_{-i}(.)=-l_{i}(.)$ are the unit vector elements that depend on the spreads of $\vec{k_{s}}(x,y)$ and $\vec{k_{-i}}(x,y)$, respectively. These vector elements allow us to model the dependency of each single Doppler shift in Eq. (2) to the x-related distance between any two points in the circles shown in Fig. 1(c). The higher the spreads of k vectors, the larger the radii of these circles. A Doppler distribution is made by adding all the single Doppler shifts introduced in Eq. (2) as

$$A(\frac{\omega_{D}\lambda }{2\pi V})=p_{R_{-i}}(\frac{\omega_{D}\lambda }{2\pi V})*p_{R_{s}}(\frac{\omega_{D}\lambda }{2\pi V}),$$ (3)

where * denotes the convolution operator, and $p_{R_{-i}}(.)$ and $p_{R_{s}}(.)$ are the probability density functions of the spreads of incoming and outgoing wavevectors, respectively. Thus the right-hand side of Eq. (3) represents an exact mathematical expression of individual wavevector differences $l_{s-i}(.)$ introduced in Eq. (2). The power spectral density can be calculated as^31^

$$P(\frac{\omega_{D}\lambda }{2\pi V})=A(\frac{\omega_{D}\lambda }{2\pi V})*A(\frac{\omega_{D}\lambda }{2\pi V}).$$ (4)

Then the normalized intensity autocorrelation is calculated by

$$g^{\left(2\right)}(\tau )=F^{-1}\{P(\frac{\omega_{D}\lambda }{2\pi V})\}\;,$$ (5)

where $F^{-1}\{.\}$ is the inverse Fourier transformation operator. Equation (5) is a representation of the Wiener–Khinchin theorem.^32^ Now, based on the properties of the Fourier transform,^33^ $g^{\left(2\right)}(\tau )$ can be calculated from $p_{R_{-i}}$ and $p_{R_{s}}$ as

$$g^{\left(2\right)}(\tau )={\left[F^{-1}\{p_{R_{-i}}\}\right]}^{2}{\left[F^{-1}\{p_{R_{s}}\}\right]}^{2}.$$ (6)

The temporal contrast of time-integrated speckle intensity is

$$C^{2}=\frac{2}{T}\int_{0}^{T}(1-\frac{\tau }{T}){\left|g^{\left(1\right)}(\tau )\right|}^{2}d\tau ,$$ (7)

where $g^{\left(1\right)}(\tau )=\sqrt{g^{\left(2\right)}(\tau )-1}$ is the normalized complex speckle field correlation^34^ (Siegert relation). See Sec. 1 in the Supplementary Material for the proof of Eq. (7) that also holds for spatial contrast of time-integrated intensity.^35^ Therefore, on a highly scattering object (i.e., infinite scattering coefficient), it is possible to predict the speckle contrast drop as a result of applied $\vec{v}$ when there is knowledge about $p_{R_{-i}}$ and $p_{R_{s}}$. The probability density functions $p_{R_{-i}}$ and $p_{R_{s}}$ can take several possible forms of which we consider two, namely, semicircular and Gaussian.

##### Semicircular density function of the wavevectors

**Fig. 2:**
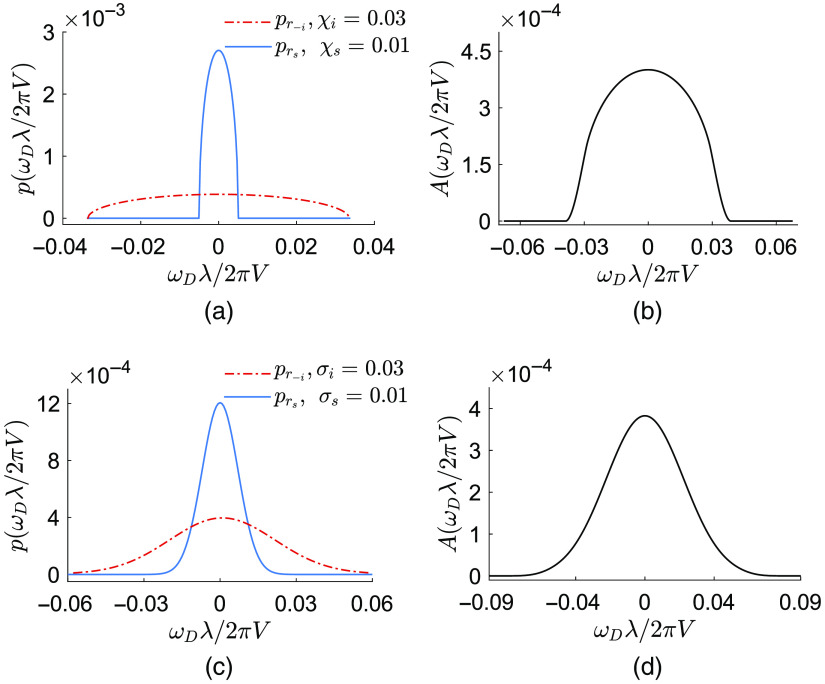
Density functions for incoming and outgoing wavevectors. λ=671  nm; V=5  mm/s. (a) Case of a semicircular shape and (b) the associated optical Doppler distribution as a result of convolution of $p_{R_{-i}}$ and $p_{R_{s}}$. (c) Case of a Gaussian shape and (d) the associated optical Doppler distribution.

We define the following form for a semicircular form of the density functions:

$$p_{-i,s}(\frac{\omega_{D}\lambda }{2\pi V})=\frac{\lambda }{\pi^{2}V\chi_{i,s}}\sqrt{1-{\left(\frac{\omega_{D}\lambda }{2\pi V\chi_{i,s}}\right)}^{2}},$$ (8)

where $\chi_{i}$ and $\chi_{s}$ are the shape parameters that control the spread of $p_{R_{-i}}$ and $p_{R_{s}}$, respectively. In Fig. 2(a), a pair of $p_{R_{-i}}$ and $p_{R_{s}}$ with semicircular shapes is represented. The corresponding optical Doppler histogram is shown in Fig. 2(b). Substituting Eq. (8) into Eq. (6) and performing the calculation results in

$$g^{\left(2\right)}(\tau )=16b_{i}^{2}b_{s}^{2}{\left(\frac{J_{1}(\frac{\tau }{b_{i}})J_{1}(\frac{\tau }{b_{s}})}{\tau^{2}}\right)}^{2}+1,$$ (9)

where $J_{1}(.)$ is the Bessel function of the first kind^36^ and $b_{i,s}=\lambda /(2\pi V\chi_{i,s})$. By substituting the calculated $g^{\left(1\right)}(\tau )$ from Eq. (9) into Eq. (7), the contrast of the time-integrated intensity takes the following form:

$$C^{2}=\frac{32b_{i}b_{s}}{T}\int_{0}^{T}\frac{1}{\tau^{4}}J_{1}{\left(\frac{\tau }{bi}\right)}^{2}J_{1}{\left(\frac{\tau }{b_{s}}\right)}^{2}[1-\frac{\tau }{T}]d\tau .$$ (10)

Equation (10) cannot be solved analytically; however, it can be calculated numerically.

##### Gaussian density function of the wavevectors

The Gaussian form of $p_{R_{-i}}$ and $p_{R_{s}}$ can be written as follows:

$$p_{-i,s}(\frac{\omega_{D}\lambda }{2\pi V})=\exp(-{\left(\frac{\omega_{D}\lambda }{2\pi V\sigma_{i,s}}\right)}^{2}),$$ (11)

where $\sigma_{i}$ and $\sigma_{s}$ are the shape parameters that control the spread of $p_{R_{-i}}$ and $p_{R_{s}}$, respectively. An example of a pair of $p_{R_{-i}}$ and $p_{R_{s}}$ with Gaussian shapes and the corresponding optical Doppler histogram are shown in Figs. 2(c)–2(d), respectively. Similar to the semicircular case, substitution of Eq. (11) into Eq. (6) results in

$$g^{\left(2\right)}(\tau )=\exp(-\frac{1}{2}a\tau^{2})+1,$$ (12)

where $a=(2\pi V/\lambda {)}^{2}[\sigma_{i}^{2}+\sigma_{s}^{2}]$. Finally, the substitution of the calculated $g^{\left(1\right)}(\tau )$ from Eq. (12) into Eq. (7) results the following contrast of the time-integrated intensity:

$$C^{2}=\frac{2}{T}[\sqrt{\frac{\pi }{2a}}\;\mathrm{erf}(T\sqrt{\frac{a}{2}})+\frac{\exp(-\frac{1}{2}aT^{2})-1}{at}],$$ (13)

where $\mathrm{erf}(z)=\int_{0}^{z}\exp(-t^{2})dt$ is the error function.

#### 2.1.2 Finite scattering medium

For a medium with finite scattering level, Eqs. (10) and (13) only hold for plane wave illumination. This is because for a finite scattering level, a single detection point B [Fig. 1(a)] is associated with a range of light injection positions A. Only for plane wave illumination, the illumination wavevector will be the same for all positions of injection of the light. Basically, with plane wave illumination, the observed Doppler shift will be independent of the light path ways from an arbitrary point A to the point B.

For the case of spherical waves, there is a single $\vec{k_{i}}$ per arbitrary point A, but the direction of each $\vec{k_{i}}$ depends on the location of A. Therefore, when illuminating with spherical or scrambled waves, information about the range of light injection positions that contribute to the detection in the observation point B is required. This information can be obtained by running a Monte-Carlo simulation of light propagation. This is beyond the scope of this paper.

### 2.2 Simulation of Time-Varying Speckle Patterns of Size 1 Pixel

For validation of the proposed theory, a time-dependent speckle complex field is generated for each pixel at location (x,y) as

$$E(x,y,t)=\frac{1}{\sqrt{N}}\overset{N}{\underset{n=1}{\sum }}\;\exp(i(\omega_{n}(x,y)t+\varphi_{n}(x,y))),$$ (14)

where N is the number of phasors set as 50 in the simulation, $j=\sqrt{-1}$, $\phi_{n}(x,y)$ is a uniformly distributed random number on the interval [−π,π] at each position (x,y), and $\omega_{n}(x,y)$ is an angular frequency picked from the optical Doppler density function $A(\omega_{D}\lambda /(2\pi V))$ introduced in Eq. (3) in a weighted manner: the higher the amplitude of frequency in $A(\omega_{D}\lambda /(2\pi V))$, the higher the chance of selecting that frequency as $\omega_{n}(x,y)$. In the dynamic speckle pattern obtained in this manner, each speckle occupies 1 pixel. Figure S1(a) in the Supplementary Material shows a simulated frame of the absolute field of size 100×100  px. The corresponding speckle intensity is calculated as [see Sec. 3 and Fig. S1(b) in the Supplementary Material]

$$I(x,y,t)={\left|E(x,y,t)\right|}^{2}.$$ (15)

The time-integrated speckle intensity is

$$I_{T}(x,y)=\int_{0}^{T}I(x,y,t)dt,$$ (16)

where T denotes the exposure time. Finally, the spatial contrast of the time-integrated speckle intensity is calculated as

$$C=\frac{\sigma_{IT}(x,y)}{\overset{¯}{I_{T}}(x,y)},$$ (17)

where $\sigma_{I_{T}}(x,y)$ and $\overset{¯}{I_{T}}(x,y)$ represent the spatial standard deviation and the mean of $I_{T}(x,y)$, respectively.

### 2.3 Imaging Geometry

**Fig. 3:**
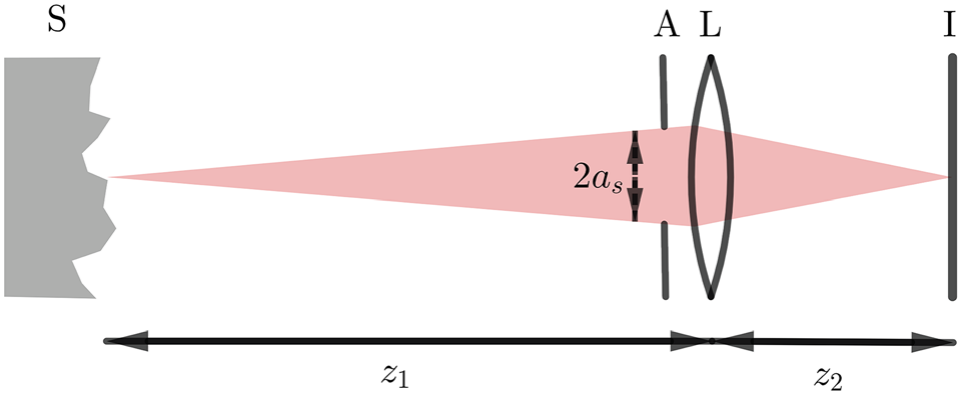
Schematic drawing of the single-lens imaging system. S, scattering medium; A, aperture; L, spherical lens; I, image screen; $z_{1}$, distance from the scattering medium to the lens; $z_{2}$, distance from the lens to the image screen; and $a_{s}$, radius of the aperture opening.

We consider the diffraction of a single-lens imaging system in order to generate speckle patterns that simulate those obtained during experiments. To be specific, a value for the shape parameters χ and σ [introduced in Eqs. (8) and (11), respectively] based on the experimental setup is determined in order to simulate dynamic speckle patterns. This way, the proposed theory is evaluated using both simulation and experiments. In this study, the scattered light is collected by a single-lens imaging system consisting of an aperture, a spherical lens, and an image screen (see Fig. 3). The surface of the scattering medium is located at the object plane. The aperture and the spherical lens are located at the aperture plane. The image screen is located at the image plane. See Sec. 2 in the Supplementary Material for description of the transfer functions.

**Fig. 4:**
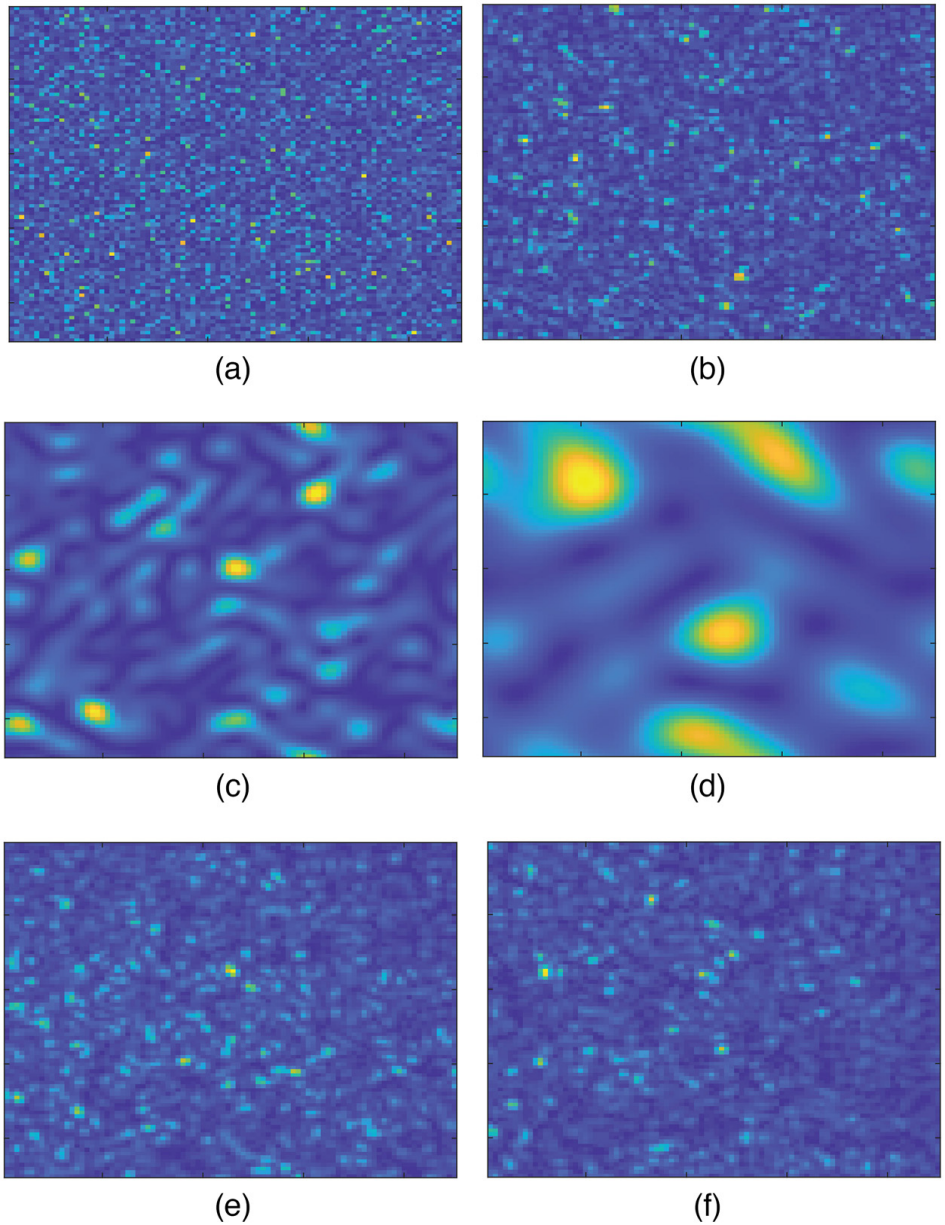
(a)–(d) Simulation of speckle intensity on the image plane of a single-lens imaging system. The speckle frame size is 90×90  px. Case of (a) no aperture (Video 1, mp4, 9.94 MB [URL: https://doi.org/10.1117/1.JBO.28.4.046005.s1]), a square aperture of (b) 46×46  px (Video 2, mp4, 9.59 MB [URL: https://doi.org/10.1117/1.JBO.28.4.046005.s2]), (c) 12×12  px (Video 3, mp4, 8.26 MB [URL: https://doi.org/10.1117/1.JBO.28.4.046005.s3]), and (d) 4×4  px (Video 4, mp4, 8.54 MB [URL: https://doi.org/10.1117/1.JBO.28.4.046005.s4]). For the sake of simplicity, a square aperture is used instead of a circular aperture. Experimental speckle intensities obtained on (e) a matte surface (Video 5, mp4, 17.5 MB [URL: https://doi.org/10.1117/1.JBO.28.4.046005.s5]) and (f) a Delrin plate (Video 6, mp4, 18.1 MB [URL: https://doi.org/10.1117/1.JBO.28.4.046005.s6]). See Sec. 3.2 for the experimental parameters. In all cases, the applied translational speed is V=10  mm/s.

A representation of simulated speckles in an imaging system with different aperture sizes for a time interval of 10 ms is provided in Figs. 4(a)–4(d) in which the wavevector density functions are circular (see Sec. 3.2 for the distances and Sec. 4 for the values of $\chi_{i}$ and $\chi_{s}$) and the applied translational speed is V=10  mm/s. These speckles are first generated pixel-by-pixel based on Eq. (14). Then the transfer functions described in Sec. 2 in the Supplementary Material are applied to them. Note that (S18) and the first term of (S19) in the Supplementary Material only apply phase shift to the speckle fields and the second term of (S19) in the Supplementary Material controls the speckle size. Therefore, it is also possible to directly generate them on the image screen and apply the spatial Fourier filter to account for the pupil function. This is equivalent to a single-lens imaging system that is considered for generation of speckles in the simulation part of this study. As a comparison with experimental speckle intensities, see Figs. 4(e) and 4(f), in which the speckle patterns obtained on a matte surface and a Delrin plate is shown, respectively. The experimental parameters for these two patterns are provided in Sec. 3.2. The speckle diameter obtained by spatial autocorrelation of intensities for Figs. 4(e) and 4(f) is about 3 pixels (each image pixel represents 63  μm), which is closest to the diameter of simulated speckles shown in Fig. 4(b), namely around 2 pixels. Note that the goal of speckle simulations is to predict temporal fluctuations of the speckles obtained via experiments rather than the speckle size. This is because it is the temporal fluctuations of the speckles that eventually govern the contrast of time-integrated intensity but not the speckle size. And since the opening of the pupil and the distance from the surface to the imaging system have been considered in the wavevector density function of the incoming waves, speckles of size 1 pixel contain all the optical Doppler shifts introduced in Eq. (1).

### 2.4 Correlation Functions

The absolute field correlation function of the stack of simulated speckles was calculated by^37^

$$g^{\left(1\right)}(\tau )=\frac{E_{1}E_{2}^{*}}{\sqrt{\left\langle {\left|E_{1}\right|}^{2}\rangle \langle {\left|E_{2}\right|}^{2}\right\rangle }},$$ (18)

where $E_{1}$ and $E_{2}$ are spatial complex fields at times $t_{1}$ and $t_{2}$, respectively. The symbols * and ⟨ ⟩ are the complex conjugate and spatial averaging operators, respectively. The time lag corresponding to the frame with a mutual correlation of 1/e with respect to a reference frame was determined as the field correlation time ($\tau_{c}$). For the speckle patterns obtained by the experiments, the mutual intensity correlations $g^{\left(2\right)}(\tau )$ were calculated as

$$g^{\left(2\right)}(\tau )=\frac{\left\langle I_{1}I_{2}\right\rangle }{\left\langle I_{1}\rangle \langle I_{2}\right\rangle }.$$ (19)

where $I_{1}$ and $I_{2}$ are the spatial intensities at times $t_{1}$ and $t_{2}$, respectively.

## 3 Materials and Methods

### 3.1 Scattering Media

In the experimental setup of this study, two static scattering media, namely a matte surface and a Delrin plate of 11-mm thickness, have been used. To prepare the matte surface, a black metal plate was painted 6 times with the intervals of 20 min with Chalk spray (Vintage) of ultra-matte color. The reduced scattering coefficient ($\mu_{s}^{\prime }$) of matte is assumed to be infinite.

### 3.2 High-Speed Imaging

A continuous wave laser light (CNI MSL-FN-671) of λ=671  nm and a coherence length of longer than 50 m was used to illuminate the scattering media. The laser beam was diverged using a planoconcave lens (Qioptiq, N-BK7, F=−6) to realize spherical waves illumination 15 cm away from the scattering medium with a power of 43.8 mW and a beam full-width-at-half-maximum of 15.6 mm.

The single-lens imaging system was made of a hard coated bandpass filter (Edmund Optics) with a wavelength of 675±12.5  nm to minimize background noise, a linear polarizer (Thorlabs, LPNIRE100-B) with the direction perpendicular to polarization of laser light to minimize specular reflection, a circular aperture with the diameter of $2a_{s}=0.8\;\mathrm{mm}$, a planoconvex lens (Thorlabs, LA1027-A) of f=3.5  cm, and a high-speed camera (Photron, Fastcam SA3) operated with a frame rate of 10 kHz. The distance from the lens to the camera sensor was set to $z_{2}=34.3\;\mathrm{mm}$ so that a sharp image was obtained when the matte and Delrin media were located at $z_{1}=15.1$ and $z_{1}=15.4\;\mathrm{cm}$, respectively, from the imaging lens. Here the resulted Fresnel numbers are $N_{F}=1.6$ and $N_{F}=1.5$ for matte and Delrin, respectively, indicating that the system is in near-field regime.

The system magnification M (i.e., the fraction of the image size of an object) has been measured as $\frac{1}{4}$. The numerical aperture of the system ($\mathrm{NA}=a_{s}/z_{2}$ in air with unity refractive index) is calculated as 0.01. Translational displacement of the scattering medium was realized by mounting them on a linear stage (Zaber, X-LHM200A-E03) perpendicular to the incoming laser beam. Note that the linear stage has a maximum permissible load of 3 kg, which is lower than the mass of the high-speed camera, which is about 6 kg. Therefore, the only option is to move the scattering media mounted on the linear stage.

### 3.3 Low-Framerate Imaging

Apart from a high-speed camera, a low-framerate camera meant for a typical LSCI was used to record speckle patterns. For that, an in-house handheld probe was mounted on the Zaber stage in order to apply translational speeds up to 30  mm/s with an acceleration of $3\;\mathrm{mm}/s^{2}$. The matte and Delrin plates were used as the scattering media in this setup. A single-mode optical fiber (Thorlabs SM600, NA=0.12) was used to couple the laser light. The $1/e^{2}\;$ beamwidth at the distance of 20 cm from the fiber tip was measured as 3 cm. A pair of planoconcave and achromatic doublet lenses (f=6  cm) were used to make a collimated beam at the distal end of the optical fiber on the probe. To make scrambled waves, the pair of lenses was replaced by a 20 deg top hat diffuser (Thorlabs EDI-s20-MD).

A monochrome camera (Basler acA2040 55um USB3) at the detection side operated with a framerate of 50 Hz and an exposure time of T=10  ms. The f-number of N=8 for the camera objective (FUJINON HF16XA-5M) with a focal length of f=16  mm was set. We calculate its aperture radius as^38^

$$a_{s}=\frac{f}{2N},$$ (20)

which equals to $a_{s}=1\;\mathrm{mm}$. Thus the Fresnel number would be $N_{F}=7.4$ indicating that the system is in the near-field regime. Similar to the high-speed imaging setup, a linear polarizer and a bandpass filter were mounted on the camera objective. Here the magnification was measured as M=0.1. To calculate spatial speckle contrast introduced in Eq. (17), a window of 90×90  px was chosen at the center of scattering spot. This low-framerate LSCI configuration is lighter than 1 kg enabling us to mount it on the linear stage to apply translational motions.

## 4 Results

In this section, the proposed model in Sec. 2 has been evaluated and validated using simulations and experiments. The specific parameters of interest are intensity correlation function and contrast of the time integrated speckle intensity. The shape parameters in the speckle simulation are tuned based on the used setups, namely high-speed and low-framerate imaging, so it enables comparing theoretical, experimental, and simulation results. The aperture size is kept constant during this study and since for a one-sided Doppler shift holding paraxial approximation $\Delta \omega_{D}=2\pi Va_{s}/\lambda z_{1}$, we define $\{\sigma_{s},\chi_{s}\}=a_{s}/z_{1}$. For the high-speed imaging setup, this equals to $\{\sigma_{s},\chi_{s}\}=26.5\times 10^{-4}$ and $\{\sigma_{s},\chi_{s}\}=26\times 10^{-4}$ for matte and Delrin, respectively. For the low-framerate imaging setup, we have $\chi_{s}=5\times 10^{-3}$ since only the semicircular model of the wavevector density functions are used to simulate speckles.

For the high-speed imaging setup, only spherical waves were used for illumination; therefore, $\{\sigma_{i},\chi_{i}\}=0$. For the low-framerate imaging setup, planar waves were used for illumination on both matte and Delrin; spherical and scrambled waves were used for illumination of matte only. Thus $\chi_{i}$ equals zero for both planar and spherical wave illumination schemes. For the scrambled wave illumination on matte, we calculate $\chi_{i}$ based on the illuminated area on the diffuser by the fiber tip. Assuming d the distance from the fiber tip to the diffuser, $\chi_{i}=d\;\mathrm{NA}/z_{1}$. We have performed this experiment for d={13.5,36,56.1}  mm that results in $\chi_{i}=\{8.1,21.6,33.7\}\times 10^{-3}$, respectively. For the aforementioned combination of illumination types, the optical Doppler histogram was calculated based on Eq. (3).

### 4.1 Intensity Correlation Functions

**Fig. 5:**
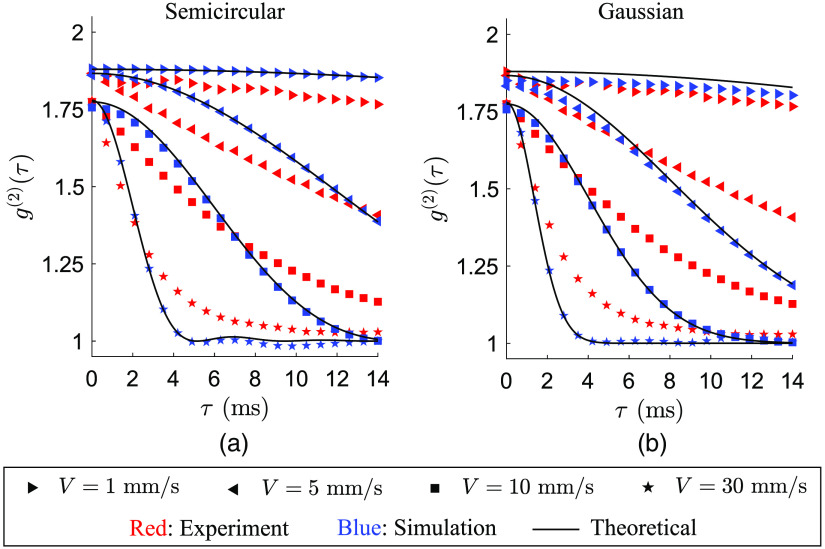
Comparison between results of simulated time-varying fully dynamic speckle patterns, experiments and the theory for a high scattering medium (matte). The theoretical curves are obtained by Eqs. (9) and (12) for semicircular and Gaussian wavevector density functions, respectively. Normalized intensity autocorrelations with the (a) semicircular and (b) Gaussian forms of the wavevector density functions. The wavevector density functions for theoretical and simulation data are made by $\{\chi_{i},\sigma_{i}\}=0$ and $\{\chi_{s},\sigma_{s}\}=26.5\times 10^{-4}$ (Video 7 mp4, 2.37 MB [URL: https://doi.org/10.1117/1.JBO.28.4.046005.s7]; Video 8, mp4, 4.26 MB [URL: https://doi.org/10.1117/1.JBO.28.4.046005.s8]).

A simulated speckle pattern calculated using Eq. (15) is shown in [Fig. S1(b) in the Supplementary Material and Videos 7 and 8], which is made by a pair of semicircular density functions introduced in Eq. (8) with $\chi_{i}=0.03$ and $\chi_{s}=0.01$. Note that speckles are simulated directly on the detector and without Fourier filtering as the spatial diffraction (the size of the simulated speckles) does not influence the contrast of the time integrated speckles. Video 7 and Video 8 show the speckle intensity frames during an episode of T=10  ms for V=2  mm/s and V=10  mm/s, respectively, and the corresponding normalized intensity correlation functions $g^{\left(2\right)}(\tau )$. Here the higher the velocity is, the more dynamic the speckle patterns are, and the more rapid the decay of the intensity correlation will be. In Fig. 5(a), $g^{\left(2\right)}(\tau )$ versus time lag is shown for a range of applied speeds as well as the theoretical predictions numerically calculated from Eq. (9), which is made by semicircular wavevector density functions for both illumination and detection. The experimental data have been shown in this graph as well, which is carried out using the high-speed imaging setup by spherical illumination on the matte surface.

Similarly, dynamic speckles are simulated using Gaussian wavevector density functions, for a range of applied velocities. The calculated intensity correlation functions versus time lag are shown in Fig. 5(b), in which the theoretical $g^{\left(2\right)}(\tau )$ curves are analytically calculated from Eq. (12). It can be observed that for both semicircular and Gaussian cases, the simulated values are in agreement with the theoretical predictions while there are deviations with the experimental data. Such deviations seem to be less severe in the semicircular case.

### 4.2 Speckle Contrast

**Fig. 6:**
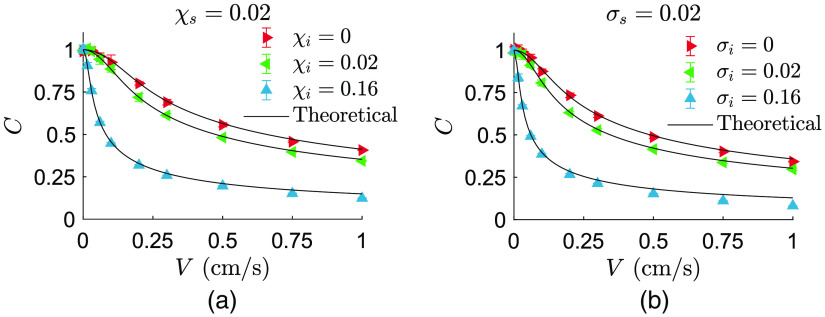
Speckle contrast (simulations) versus the applied translational speed. The density functions for the wavevectors are (a) semicircular and (b) Gaussian. $\{\chi_{i},\sigma_{i}\}=0$ simulates spherical waves on matte and planar waves on both matte and Delrin, whereas $\{\chi_{i},\sigma_{i}\}>0$ simulates scrambled waves on matte.

In a typical LSCI system, the usual measure of the speckle dynamics is spatial contrast introduced in Eq. (17). In Fig. 6(a), the spatial speckle contrast is shown versus the applied translational speed for semicircular density functions introduced in Eq. (8). The theoretical curves are calculated based on Eq. (10), whereas the simulated points are obtained by Eq. (17). Here the detection system (modeled by $\chi_{s}$) is kept constant, whereas three different values for the spread of illumination wavevectors (i.e., $\chi_{i}$) are realized. $\chi_{i}=0$ simulates planar or spherical waves, whereas $\chi_{i}>0$ simulates scrambled waves such that a larger $\chi_{i}$ corresponds to a wider area on a diffuser’s surface being illuminated.^30^ Similarly, in Fig. 6(b), the spatial speckle contrast is plotted versus the applied translational speed for Gaussian density functions shown in Eq. (11). The theoretical curves are obtained by Eq. (13). The effect of illumination types is simulated here by changing $\sigma_{i}$ while keeping $\sigma_{s}$ unchanged.

**Fig. 7:**
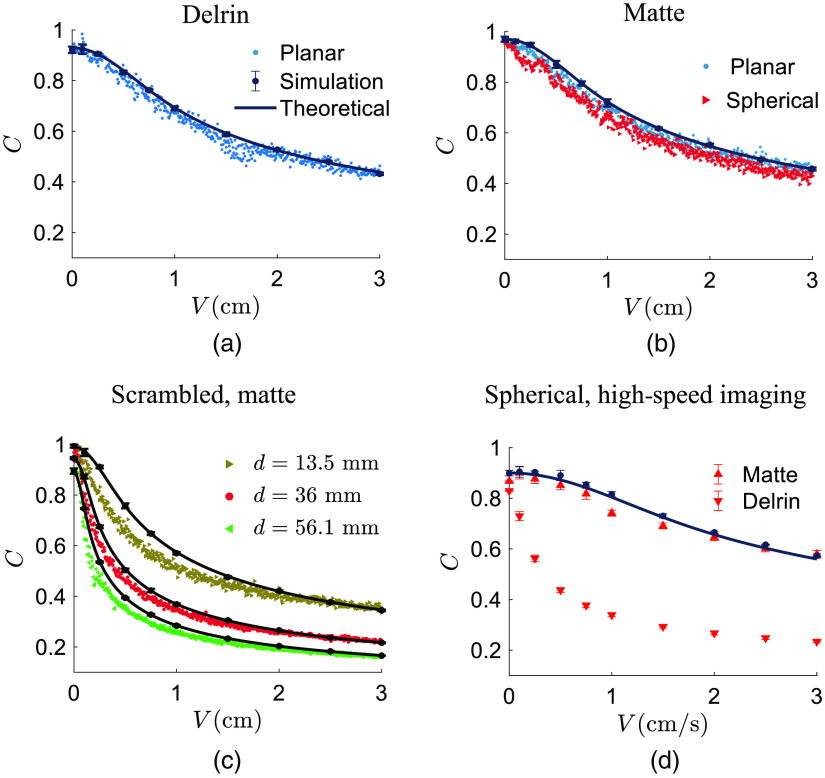
Experimental results of the contrast of the time-integrated speckle intensities versus applied translational speed obtained by (a)–(c) low framerate and (d) high-speed imaging setups. Solid curves with the accompanying data points correspond to theoretical and speckle simulation in all subfigures. Data points with error bars represent mean ± standard deviation.

In Fig. 7, experimental results of the contrast of the time integrated speckles for different illuminations and media types are depicted versus the applied translational speed. Notice that Figs. 7(a)–7(c) correspond to the low framerate, whereas Fig. 7(d) corresponds to the high-speed imaging setups.

The case of scrambled wave illumination on a matte surface is shown in Fig. 7(c) for three distance of fiber tip to diffuser d where for a certain speed, the higher the distance, the more the speckle contrast drop is obtained. Here the theoretical curves slightly deviate the corresponding experimental data points for smaller speeds. Finally, in the high-speed imaging setup, an amount of 100 consecutive speckle intensity frames were averaged to realize an exposure time of T=10  ms.

The pair of planar wave illumination on Delrin is depicted in Fig. 7(a), where the experimental data points fairly match the theoretical prediction and simulation. In Fig. 7(b), the two illumination types of planar and spherical waves are shown where the matte surface is used as the scattering medium. The theoretical curves in Figs. 7(a) and 7(b) are the same, and it is expected to obtain the same experimental data for these cases. Then their spatial contrasts were calculated according to Eq. (17) to form the experimental data points shown in Fig. 7(d) versus the applied translational speed for matte and Delrin. For a certain speed, the speckle contrast on Delrin is lower than the speckle contrast calculated on matte. The theoretical curves in Fig. 7 have been numerically calculated using Eq. (10).

## 5 Discussion

A theoretical model is proposed to predict MA caused by translation of a handheld LSCI system, for the case of fully static media. The model step by step works back from the speckle contrast to its origins in the optical Doppler shifts, and to the role of the wavevectors of the illumination and the detection that govern these Doppler shifts. Semicircular and Gaussian forms are proposed for the density functions of the incoming and outgoing wavevector sets. Analytical expressions for the contrast of time-integrated intensity for the two types of density functions have been derived that can be calculated numerically.

Equation (7) suggests that the speckle contrast drop is independent of the type of speckle dynamics in terms of boiling, shifting, or a combination of both. This is because in the assumptions of the steps to derive Eq. (7) discussed in Sec. 1 in the Supplementary Material, no discrimination was necessary between boiling or shifting of the speckle movements and the fact that fully developed speckle patterns share equivalent temporal and spatial correlation functions. What dictates the speckle contrast drop is the spatial one-point autocorrelation of the field. This statement is not concluded by the developed model but it is purely originated from the definition of speckle contrast. The importance of this statement is that it facilitates further development of the model.

Prediction of speckle intensity correlation and time-integrated speckle contrast was assessed by simulation of dynamic speckles where an agreement has been found between the simulated and the theoretical values for the cases of semicircular and Gaussian density functions (Figs. 5 and 6). These results are applied for translation of the system when illuminating a high scattering surface with planar, spherical, or scrambled waves as well as translation of the system when illuminating a finite scattering medium with planar waves. For the cases of spherical or scrambled wave illumination on a finite scattering medium, Monte Carlo simulation will be needed to obtain the fluence distribution around each detection point at the surface as an input for the calculation of the optical Doppler distribution $A(\omega_{D}\lambda /(2\pi V))$. This forms a crucial topic for the future research.

The experimental study was carried out in two setups, namely, a high-speed and a low-framerate imaging system. In the former, a high scattering surface (matte) and a finite scattering medium (Delrin) were illuminated by spherical waves. Intensity correlation and spatial contrast of the recorded speckle patterns were studied [Figs. 5 and 7(d)]. In the latter, three scenarios were studied: planar wave illumination on Delrin, planar, and spherical wave illuminations on matte and scrambled wave illumination on matte [see Fig. 7(a)–7(c)]. It has been observed that planar wave illumination results in the same contrast–speed curve independent of the media [Fig. 7(a) and 7(b)]. This is in agreement with our previous experiments.^27^ In this study, we have shown that the proposed model can accurately predict, such speckle contrast drop. Also for the same applied speed, a longer diffuser distance to the fiber tip causes more speckle contrast drop due to the role of incoming wavevectors in creation of Doppler shifts [Fig. 7(c)]. Moreover, for the same applied speed, Delrin causes more drop in speckle contrast compared to matte, which is due to the optical wave propagation in the media and the incoming wavevectors being location dependent when using scrambled or spherical waves for illumination [Fig. 7(d)].

The essence of intensity correlation analysis provided in Fig. 5 is first to evaluate theoretical predictions of correlation time derived in Eqs. (9) and (12) by speckle simulation and experiments and second to make a comparison between semicircular and Gaussian forms of the wavevector density functions. As for the theoretical and simulation curves, one might argue that it is the circular reasoning that causes the precise agreement of the corresponding curves because what has been injected as Doppler shift will eventually be extracted in the output. Although both theoretical and simulation cases share the same wavevector density functions, the theoretical curves are obtained directly by calculating Eqs. (9) and (12) and the simulation data are a result of the generation of time-varying speckle intensities and the calculation of their correlation functions based on Eq. (19).

Thus the corresponding pairs would deviate if Eqs. (9) and (12) were derived incorrectly. The same discussion holds for the speckle contrast data shown in Fig. 6, in which the validity of Eqs. (10) and (13) have been examined by calculating contrast of simulated speckles using Eq. (17). As for the comparison between semicircular and Gaussian forms of the wavevector density functions, it is apparent that the semicircular slightly outperforms the Gaussian when it comes to intensity correlation functions (Fig. 5). Therefore, the semicircular form has been used to further calculate the speckle contrast and compare the cases of spherical illumination on matte and Delrin (Fig. 7).

The mismatch between theoretical/simulation and experimental results of intensity correlation obtained in Fig. 5 can be due to experimental imperfections, such as imperfect focus of the lens, aberration, and error in calculation of distances, but also the fact that neither semicircular nor Gaussian are the perfect functions to model the intensity correlation. However, such mismatch becomes negligible when calculating the contrast of time-integrated speckles shown in Fig. 7(d) for the case of Matte. To explain this, we should take into account the weighting factor 1−τ/T in Eq. (7). This factor decreases the effect of $g^{\left(1\right)}(\tau )$ as τ increases especially for the values shorter than 10 ms. Therefore, the simulated and experimental speckle contrast values calculated from this integral will have more agreement.

Although the theoretical/simulated $g^{\left(2\right)}(\tau )$ start from the value of 2, the experimental curves in Fig. 5 start from lower values. On this basis, the starting points of the theoretical/simulated curves are tuned based on the experimental curves. Such imperfection in the experimental results is due to the specular reflection from the surface, which is minimized using a polarizer but not completely removed. This results in detection of high-intensity values at random locations of a speckle pattern that may not saturate the camera sensor but makes the phase function $\phi_{n}(x,y)$ introduced in Eq. (14) to have a different statistical distribution than uniform. As a result, the so-called partially developed speckle patterns are observed (as opposed to fully developed speckle patterns) and the calculated intensity correlation introduced in Eq. (19) at time lag τ=0 has a value lower than 2. It is also observed in Fig. 5 that the curves of higher speed are of lower $g^{\left(2\right)}(0)$. Here the reason is the vibration of the linear stage applied to the pair of illumination-detection system and scattering medium. As the motor rotates faster, more vibrations are made, which causes detection of more dynamic speckle intensities.

We initialized the study of the influence of the spread of $\vec{k_{i}}$ on the occurring Doppler shifts and the consequent speckle contrast drop using a diffuser for illumination in Ref. 30. In LSCI systems, diffusers are the most common devices used for illumination. In this work, we have studied the influence of $\vec{k_{i}}$, $\vec{k_{s}}$, and $\vec{v}$. We considered single-lens imaging in the experimental setups of this study, which is the most straightforward case to model. Modeling an imaging system enables connecting the experimental parameters, such as aperture size and distances to the scattering surface and image screen to the factors embedded in the model namely the shape parameters. This way, the model is fine-tuned to predict the speckle contrast drop in a system with specific experimental parameters. The other reason to consider the imaging system is to verify that it falls under the Fresnel diffraction by calculating its Fresnel number introduced in (S20) in the Supplementary Material. In this case, the transfer function of the imaging system consists only of the two terms introduced in (S18) and (S19) in the Supplementary Material, which suggests that the speckle correlation function is not influenced by the spatial diffraction. This feature simplifies the procedure of time varying speckle simulation by enabling generation of speckles of size 1 pixel directly on the image screen. Essentially, what control the correlation functions of the speckles are applied speed and the incoming and outgoing wavevector density functions. In Ref. 39, we have employed the model of the current study to predict speckle contrast drop in a handheld LSCI system in which the on-surface speeds have been measured by a vision-based technique in a dual-camera setting.

Throughout this work, only translation of the LSCI system has been studied while in practice, rotation and tilt of wavefront also play role in creation of MA. Also in an *in vivo* LSCI measurement, the object of interest is human skin, which is dynamic and of finite scattering level. Therefore, it is essential to expand the model to include dynamic media in which some extent of movements including Brownian motion is introduced and that the rotation and tilt of wavefronts are taken into account. In that case, the proposed model should be adjusted and the validation simulation and experiments should be performed.

## 6 Conclusion

A model has been developed with the purpose of predicting MA caused by translation in handheld laser speckle contrast perfusion imaging. Our model is based on the optical Doppler shift distributions associated with the range of wavevectors for illumination and detection. It provides a quantitative description of an already introduced influence of illumination, detection, and applied speed on the MA in an LSCI system (Fig. 1).

The model has been validated by simulation of dynamic speckles as well as by experiments with a high-speed camera equipped with a single-lens imaging system as well as a low-framerate camera typical for an LSCI system with a camera objective. The model validation consisted of temporal analysis using intensity correlation functions (Fig. 5) and spatial analysis using contrast of the time-integrated speckle intensity (Figs. 6 and 7). We have concluded that although the model fails to exactly predict the intensity correlation functions, it successfully predicts contrast of the time-integrated speckle when measuring on both a high scattering surface and a tissue mimicking scattering medium (Delrin).

Results of this study help to advance model-based prevention and/or correction of MA occurring during a handheld LSCI measurement. Realization of a handheld LSCI system is crucial in assisting medical staff especially during surgery, in which compactness, speed, and reliability of the measurement system are of pronounced importance.

## Code, Data, and Materials Availability

The dataset generated during this study can be found in dataset 1.^40^ All the materials used for experiments are in possession of the University of Twente and can be used for the purpose of reproducibility after obtaining permission from the University of Twente.
