# Supplementary material for: Prediction of motion artifacts caused by translation in handheld laser speckle contrast imaging
Source: J Biomed Opt. 2023 Apr 18;28(4):046005. doi: 10.1117/1.JBO.28.4.046005 (PMC10112282; doi:10.1117/1.JBO.28.4.046005)
Supplement: Supplementary file 1 [file JBO_028_046005_SD001.pdf]

Supplemental material for prediction of motion artifacts caused by translation in handheld laser speckle contrast imaging

Ata Chizari[a,b,*], Wilson Tsong[a,b], Tom Knop[a], and Wiendelt Steenbergen[a]

[a] Biomedical Photonic Imaging group, Technical Medical Centre,
Faculty of Science and Technology, University of Twente, PO Box 217,
7500 AE Enschede, The Netherlands
[b] These authors contributed equally to this work
*a.chizari@utwente.nl

1 Contrast of the time-integrated intensity for a fully dynamic speckle

The equation that connects the contrast of fully dynamic speckle patterns to the integration of the field or intensity correlation is not clearly explained in the literature. The assumption is that all light undergoes a Doppler shift and the resultant speckle patterns therefore do not have a static part. Here, we employ the theory of Goodman[34] on high-order coherence of polarized thermal light (i.e. electromagnetic radiations emitted from a light source as a consequence of its temperature) to calculate the temporal speckle contrast. Since the subject speckle patterns are fully developed, they share the same statistics in space and time. Thus, the result of the analysis is nearly identical for integrals over space in order to calculate the spatial speckle contrast. Consider the instantaneous intensity $I(x, y, z, t)$ of a complex field $E(x, y, z, t)$ at a point $P(x, y, z)$ in space. The observed time-integrated intensity on a detector is

$$I_T(t) = \int_{t-T}^{t} I(\xi) d\xi, \tag{S1}$$

with T the exposure time. Since the thermal light is an ergodic random process (and therefore stationary), the statistics of $I_T(t)$ is independent of the observation time, allowing us to write

$$I_T(t) = \int_{-\frac{T}{2}}^{\frac{T}{2}} I(\xi) d\xi. \tag{S2}$$

The expected (i.e. mean) value of the time-integrated intensity is

$$\overline{I_T} = \mathbb{E}\left[\int_{-\frac{T}{2}}^{\frac{T}{2}} I(\xi)d\xi\right]$$

$$= \int_{-\frac{T}{2}}^{\frac{T}{2}} \mathbb{E}[I(\xi)]d\xi$$

$$= \bar{I}T. \tag{S3}$$

The variance of the time-integrated intensity is

$$\sigma_{I_T}^2 = \mathbb{E}[I_T^2] - \overline{I_T}^2. \tag{S4}$$

The first term (the average of the square intensity) has the form

$$\mathbb{E}[I_T^2] = \mathbb{E}\left[\iint_{-\frac{T}{2}}^{\frac{T}{2}} I(\xi)I(\eta)d\xi d\eta\right]$$

$$= \iint_{-\frac{T}{2}}^{\frac{T}{2}} \mathbb{E}[I(\xi)I(\eta)]d\xi d\eta$$

$$= \iint_{-\frac{T}{2}}^{\frac{T}{2}} \Gamma_I(\xi - \eta)d\xi d\eta. \tag{S5}$$

The function $\Gamma_I(\tau)$ is the autocorrelation function of the instantaneous intensity that is defined as

$$\Gamma_I(\tau) = \mathbb{E}[E(t)E^*(t)E(t+\tau)E^*(t+\tau)]$$

$$= \lim_{x\to\infty} \frac{1}{x}\int_0^x I(t)I(t+\tau)dt\,, \tag{S6}$$

where $*$ represents the complex-conjugate operator and $\Gamma_I(\tau)/\bar{I}^2 = g^2(\tau)$. Equation (S5) can be rewritten as

$$\mathbb{E}[I_T^2] = \iint_{-\infty}^{\infty} \mathrm{rect}(\tfrac{\xi}{T})\mathrm{rect}(\tfrac{\eta}{T})\Gamma_I(\xi-\eta)d\xi d\eta, \tag{S7}$$

where $\mathrm{rect}(x) = 1$ for $|x| \le \frac{1}{2}$ and zero otherwise. By replacing the variables $\xi = \tau + t$ and $\eta = t$ (and thus $d\xi = d\tau$ and $d\eta = dt$) we have

$$\mathbb{E}[I_T^2] = \iint_{-\infty}^{\infty} \mathrm{rect}\left(\frac{\tau+t}{T}\right)\mathrm{rect}\left(\frac{t}{T}\right)\Gamma_I(\tau)d\tau dt$$

$$= \int_{-\infty}^{\infty} \underbrace{\left[\int_{-\infty}^{\infty} \mathrm{rect}\left(\frac{\tau+t}{T}\right)\mathrm{rect}\left(\frac{t}{T}\right)dt\right]}_{f(\tau)}\Gamma_I(\tau)d\tau. \tag{S8}$$

The functions $\mathrm{rect}\left(\frac{\tau+t}{T}\right)$ and $\mathrm{rect}\left(\frac{t}{T}\right)$ return unity for $-\frac{T}{2} - |\tau| \le t \le \frac{T}{2} - |\tau|$ and $-\frac{T}{2} \le t \le \frac{T}{2}$, respectively, and zero otherwise. The intersection of the last two intervals would mean $-\frac{T}{2} \le t \le \frac{T}{2} - |\tau|$, that results in

$$f(\tau) = \int_{-\frac{T}{2}}^{\frac{T}{2}-|\tau|} d\tau = T - |\tau|. \tag{S9}$$

Substituting (S9) into
(S8) and considering $-\frac{T}{2} \le \frac{T}{2} - |\tau|$, we have

$$\mathbb{E}[I_T^2] = \int_{-T}^{T}[T - |\tau|]\Gamma_I(\tau)d\tau.$$

(S10)

The autocorrelation function $\Gamma(\tau)$ introduced in (S6) under the assumption of a Gaussian random process can be expressed as[33]

$$\Gamma_I(\tau) = \bar{I}^2\left[1 + \left|g^{(1)}(\tau)\right|^2\right], \quad \text{(S11)}$$

where $g^{(1)}(\tau)$ is the complex degree of coherence of the light, also known as the normalized complex field correlation with the following definition[39]

$$g^1(\tau) = \frac{\mathbb{E}[E(t+\tau)E^*(t)]}{\sqrt{\mathbb{E}[|E(t+\tau)|^2]\mathbb{E}[|E(t)|^2]}}.$$

(S12)

Now, substitution of (S11) into

(S10) and further simplification yields the result

$$\mathbb{E}\left[\bar{I_T}^2\right] = \bar{I}^2\ \left[\int_{-T}^{T}[T - |\tau|]|g^1(\tau)|^2 d\tau + T^2\right].$$

(S13)

Finally, the temporal speckle contrast is

$$C^2 = \frac{\sigma_{I_T}^2}{\bar{I_T}^2}$$

$$= \frac{1}{T}\int_{T}^{T}[T - |\tau|]|g^1(\tau)|^2 d\tau$$

$$= \frac{2}{T}\int_0^T \left[1 - \frac{|\tau|}{T}\right] |g^1(\tau)|^2 d\tau.$$

(S14)

In a single scattering regime and assuming Browning motion of the scattering particles, the normalized field correlation function has a negative exponential form

$$g^{(1)}(\tau) = \exp\left(-\frac{|\tau|}{\tau_c}\right), \tag{S15}$$

with τ_c the correlation time, that is the time it takes for the correlation function to reach $1/e$ of its maximum value. Substituting (S15) into

(S14) and calculating the integration will result

$$C^2 = \frac{\tau_c}{T} + \frac{\tau_c^2}{2T^2}\left(\exp\left(-\frac{2T}{\tau_c}\right) - 1\right), \tag{S16}$$

which is the conventional model for laser speckle contrast imaging (LSCI)[3,10].

2 Single-lens imaging transfer function

The complex amplitude of detected field at the image plane $U_1(x, y)$ is calculated by diffraction of the complex amplitude of scattered field from the object plane $U_0(x', y')$ traveled through the imaging system. Assuming a linear shift-invariant imaging system, the detected light in the Fourier domain is[40]

$$\mathcal{F}\{U_1(x, y)\} = \mathcal{F}\{U_0(x', y')\}H(\nu_x, \nu_y), \tag{S17}$$

where $\mathcal{F}\{.\}$ is the spatial Fourier transformation operator. (ν_x, ν_y) indicates the spatial frequency components in the $x - y$ plane and $H(\nu_x, \nu_y)$ is the transfer function of this imaging system consisting of a free-space propagation from the object plane to the aperture plane

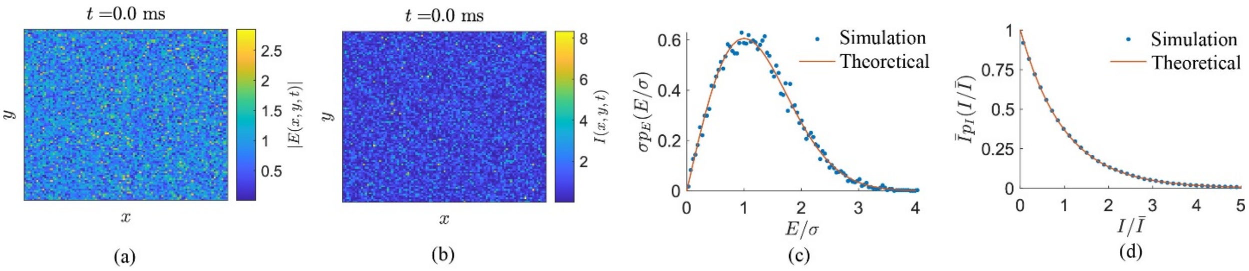

Fig. S1 Simulation of fully dynamic speckle patterns. A frame of speckle (a) absolute field and (b) the corresponding intensity. Normalized probability density functions (PDFs) of the (c) absolute field and (d) intensity as a comparison between results of simulated speckle patterns and theory.

$H_{z_1}(\nu_x, \nu_y)$ and from the aperture plane to the image plane $H_{z_2}(\nu_x, \nu_y)$ that also includes the aperture function. The transfer function corresponding to the free space propagation is[41]

$$H_{z_1}(\nu_x, \nu_y) = \exp(-jkz_1)\exp\left(-jkz_1(\nu_{x'}^2 + \nu_{y'}^2)\right), \tag{S18}$$

where $k = 2\pi/\lambda$ is the wavenumber and z_1 is the distance from the object plane to the aperture plane. The second part of the transfer function in the Fourier domain is[40]

$$H_{z_2}(\nu_x, \nu_y) = \exp(-jkz_2)p(\alpha, \beta), \tag{S19}$$

where z_2 is the distance from the aperture plane to the image plane. For a system in focus, namely $1/z_1 + 1/z_2 = 1/f$, with f the focal length of the spherical lens, $p(\alpha, \beta)$ is the spatial Fourier transform of the pupil (aperture) function. Therefore, the transfer function of the imaging system $H(\nu_x, \nu_y)$ introduced in (S17) is calculated by multiplication of the two components introduced in (S18) and (S19).

The main assumption here is that the system is aberration-free and falls under the Fresnel diffraction (i.e. near-field regime). To fulfill this, the Fresnel number N_F should be in the neighborhood of the unity and is defined as

$$N_F = \frac{a_s^2}{\lambda z_1},$$ (S20)

where a_s is the radius of aperture opening.

3 Simulation of speckle patterns of size 1 pixel

The absolute field of a fully developed speckle pattern follows a Rayleigh distribution with the following form[39] (see Fig. S1(c))

$$p_E(E) = \frac{E}{\sigma^2} \exp\left(-E^2/(2\sigma^2)\right),$$ (S21)

where $\sigma = \sigma_R = \sigma_I$ is the standard deviation of either real or imaginary part of the complex field. In addition, a fully developed speckle pattern has a negative exponential intensity distribution with the following form (see Fig. S1(d))

$$p_I(I) = \frac{1}{\bar{I}} \exp\left(-I/\bar{I}\right),$$ (S22)

where $\bar{I} = 2\sigma^2$ is the mean intensity.
